# Demonstration of Triband Multi-Focal Imaging with Optical Coherence Tomography

**DOI:** 10.3390/app8122395

**Published:** 2018-11-26

**Authors:** Ahhyun Stephanie Nam, Jian Ren, Brett E. Bouma, Benjamin J. Vakoc

**Affiliations:** 1 Wellman Center for Photomedicine, Massachusetts General Hospital, Boston, MA 02114, USA; 2 Department of Dermatology, Harvard Medical School, Boston, MA 02115, USA; 3 Division of Health Sciences & Technology (HST), Massachusetts Institute of Technology, Cambridge, MA 02139, USA

**Keywords:** optical coherence tomography, multi-focal imaging, chromatic focal shift

## Abstract

We demonstrate an extended depth of focus optical coherence tomography (OCT) system based on the use of chromatic aberration to create displaced focal planes in the sample. The system uses a wavelength-swept source tuning over three spectral bands and three separate interferometers, each of which interfaces to a single illumination/collection fiber. The resulting three imaged volumes are merged in post-processing to generate an image with a larger depth of focus than is obtained from each band individually. The improvements are demonstrated in structural imaging of a porous phantom and a lipid-cleared murine brain, and by angiographic imaging of human skin. By using a coaxial approach with Gaussian beams, this approach enables an extended focus with relatively simple microscope optics and data-merging algorithms.

## 1. Introduction

Optical coherence tomography (OCT) is an imaging modality based on low coherence interferometry [1]. Compared to other optical methods such as multiphoton and confocal microscopies, a typical OCT system operates with reduced resolution but offers larger imaging fields and deeper penetration depth in biological samples. The axial and transverse resolutions of OCT are set by the spectral bandwidth of the light source and the numerical aperture (NA) of the imaging lens, respectively. While higher NA lenses can be used to increase transverse resolution in OCT, this results in a limited depth of focus (DoF). Therefore, most systems operate with moderate transverse resolutions that approximately match DoF to the relatively long penetration depth of OCT.

Various strategies have been introduced to address the trade-off between transverse resolution and DoF. Most straightforward is the serial acquisition of imaging data at discrete focal planes followed by a merging/fusion of these imaging data into a single image or volume [2,3]. This approach, however, slows imaging speed and imposes requirements on sample stability. Systems that illuminate the sample with multiple beams, each with a distinct focal plane, allow simultaneous acquisition of multi-focal data. While simple in design, these systems have the disadvantage of reducing the power in each beam [4,5] and requiring lateral registration of the non-coaxial beams [6]. More complex approaches based on shaped (e.g., Bessel) beams have also been demonstrated [7–13]. For endoscopic imaging, shaping the axial profile of the sample beam at the end of a miniaturized fiber-optic probe [14–17] to create more axially uniform irradiance can extend the focal range of the imaging probe in a similar manner. In addition to needing more complex optics to generate the shaped beam, these methods negatively affect signal collection efficiency and generate higher side-lobes in the transverse point-spread function. Approaches involving digital refocusing of the complex field [18–20] have been reported. While effective and powerful, these methods require a sample phase-stability that is difficult to achieve in many settings.

In this manuscript, we demonstrate an approach for multi-focus imaging using triband illumination. Chromatic aberration has been used previously to extend DoF in high-resolution microscopy [21] and has applied to the fiber-optic probe design of the subcellular imaging in OCT [14]. Our system is based on the same principle; we perform simultaneous imaging in three spectral bands centered at 1060 nm, 1310 nm, and 1550 nm. We engineered the chromatic aberration of the microscope optics to cause a displacement in the focal plane of each band such that imaging data across all three bands can be merged to create a single extended-focus acquisition. This approach has the advantage of maintaining a single-fiber microscope with Gaussian beam illumination and detection, while also simplifying the fusion of the imaging data by using coaxial beams.

## 2. System Implementation

**Figure 1:**
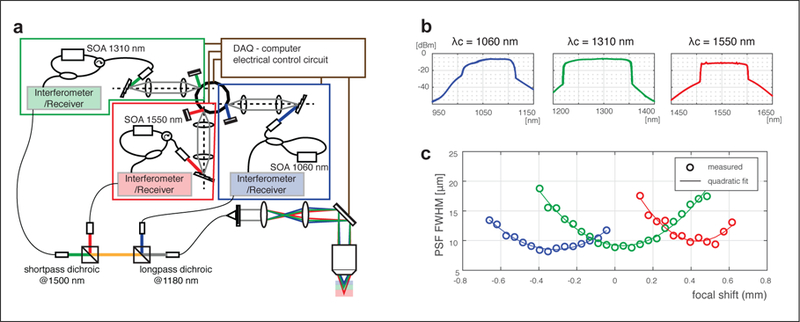
Triband system overview. (**a**) System schematic. Each band has its own interferometer and polarization-diverse receiver (not shown). (**b**) Optical spectrum of each swept source and (**c**) Measured transverse PSF as a function of depth for each band (blue: 1060 nm; green: 1310 nm; red: 1550 nm).

The OCT system generated wavelength-swept output at three spectral ranges as shown in Figure 1a. Each source comprised a semiconductor optical amplifier (SOA), a fiber-ring cavity and a swept wavelength filter [22,23]. All three sources shared a single polygon scanner in their respective swept filters, which synchronized the sweep rates across the sources to 50 kHz. The measured power spectra of the sources are given in Figure 1b. The 3 dB bandwidth of each spectral band was measured as 100 nm, 135 nm, and 98 nm at the center wavelengths of 1060 nm, 1310 nm, and 1550 nm, respectively.

Each spectral band had a dedicated interferometer with acousto-optic frequency shifter [24] and optical circuit for polarization-diverse detection (not shown in Figure 1) [25]. Polarization-sensitive detection avoids signal fading and slightly improves image quality by reducing speckle noise. The sensitivity of each interferometer was 97.5 dB, 98.7 dB and 102.3 dB for the 1060 nm, 1310 nm, and 1550 nm bands, respectively. Corning HI1060 single mode fiber was used for the 1060 nm system, and SMF28 single mode fiber was used for the 1310 nm and 1550 nm interferometers. The data from each interferometer was acquired simultaneously using three 2-channel DAQ cards (PX14400; Signatec, Lockport, IL, USA). A customized acquisition application written in C++ was developed to stream the recorded data to a RAID hard-drive array. Light from the sample arms of each interferometer was combined into a single HI1060 microscope fiber using free-space dichroic beam splitters (Thorlabs DMLP1180, DMSP1500). The optical power incident on the sample was 2.0 mW, 3.5 mW, and 2.2 mW and the axial resolution was measured to be 7.72 µm, 8.12 µm, and 10.08 µm for the 1060 nm, 1310 nm, and 1550 nm bands, respectively.

We used optical design software (Optics Studio; Zemax LLC, Kirkland, WA, USA) to design the chromatic aberration of the microscope optics. A 10X OCT scan lens (LSM02; Thorlabs, Newton, NJ, USA) was used as an imaging objective, before which two lenses (49–116; Edmund Optics, Barrington, NJ, USA) were inserted in infinite conjugate configuration to induce additional chromatic shift. The achieved displacement of the focal planes was confirmed experimentally by measuring the transverse point-spread function (PSF) using 5 µm microspheres (4K-05; Thermo Fisher Scientific, Waltham, MA, USA) dispersed in an agarose gel. At a given depth plane, the microspheres were manually located, and the PSF diameter was measured as the full width half maximum (FWHM) of the peak amplitude. The median of the PSF width is plotted as a function of depth in Figure 1c. The induced focal shifts relative to the 1310 nm band was calculated by fitting the measured PSFs to a quadratic. The shifts of the 1060 nm band and 1550 nm bands (relative to 1310 nm) were 0.352 mm and 0.396 mm, respectively.

## 3. Registration and Fusion of Imaging Data

The measurements from each band were combined to generate a single image with extended DoF. Because each band has its own delay location, axial resolution, and axial sampling (i.e., pixel spacing), a registration and fusion procedure was developed using a polyurethane foam sponge as a reference sample. To normalize the axial sampling, we empirically determined the depth range of each band’s individual images, and from this derived the appropriate zero-padding to use in subsequent DFTs of each band to result in equal axial spacing. Here we note that a separate numerical dispersion compensation was applied to each band to remove any dispersion mismatch between the sample and reference arms. The axial location of a common structure was measured in each band to align the axial positions of each dataset. Finally, we merged the in-focus imaging data from each band at that band’s native axial resolution, resulting in an image with slightly non-uniform axial resolution. It would also be possible to generate an image with a constant axial resolution by downgrading the axial resolutions of the 1060 nm and 1310 nm bands to match that of the 1550 nm.

**Figure 2:**
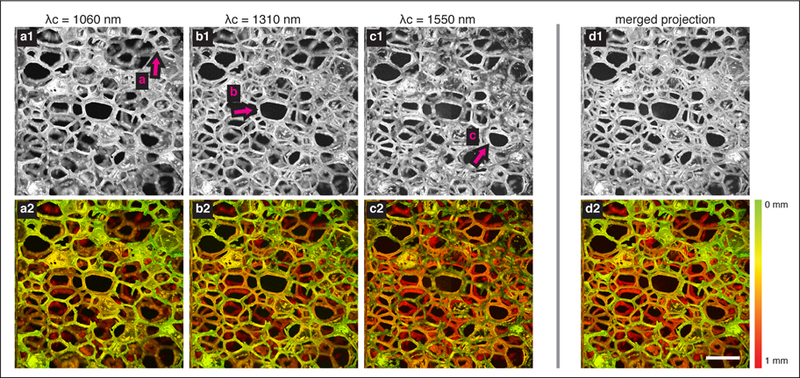
Projection images of polyurethane sponge. (**a**–**c**) MIP of the imaged volume from the individual interferometers where the source spectrum is centered at 1060 nm, 1310 nm, and 1550 nm, respectively. The arrows (a–c) highlight the in-focus depth in the imaging field. (**d**) MIP of the merged volume. The first row shows MIPs in grayscale, and the second row shows colorized MIPs with pseudo-colormap encoding the depth. Scale bar = 1 mm.

The result is presented in Figure 2. The depth-encoded maximum intensity projection (MIP) was created generating a Hue-Saturation-Value image with the depth of the MIP signal mapped to Hue ([0, 0.4]), the MIP signal (after normalizing to the range [0, 1]) mapped to Value, and the Saturation set uniformly to 1.0. The projections applied to individual imaging bands are shown in Figure 2a–c. The magenta arrows mark locations in which the sponge fibers are in focus. The projection of the extended-focus dataset is shown in Figure 2d and demonstrate higher resolution imaging across the entirety of the 1 mm depth range.

## 4. OCT Angiography of Human Skin

**Figure 3:**
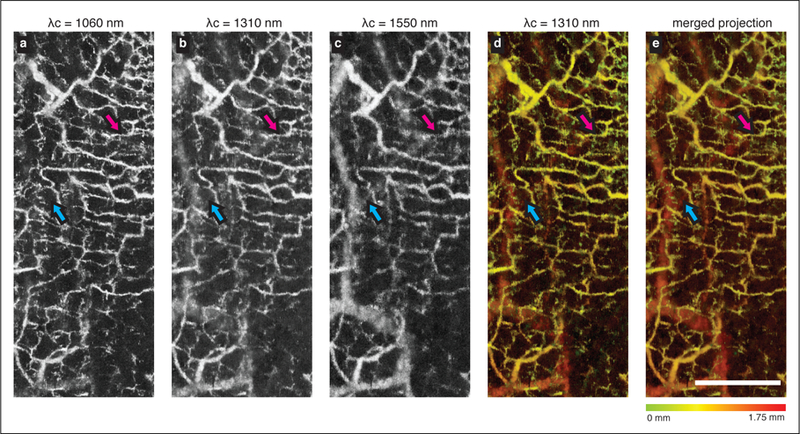
The vascular projections acquired from the bands centered at (**a**) 1060 nm, (**b**) 1310 nm, and (**c**) 1550 nm are shown. Projections were obtained using a colormap to encode the depth of the vessel, in (**d**) imaging band at 1310 nm, and (**e**) combining all three imaging bands. The magenta arrows mark the superficial vessel that is best captured by the 1060 nm system. The blue arrows mark the deeper vessel that is best captured by the 1550 nm system. Scale bar = 1 mm.

The extended-focus imaging can be used to better resolve smaller vessels in OCT angiography acquisitions across substantial depth ranges [26]. To demonstrate this, we imaged the microvasculature in human skin on the palm of a healthy volunteer. A scanning protocol providing 7 measurements at each of 1184 transverse locations in the X-axis was used. The vascular contrast was computed using previously described complex differential variance algorithm that has high flow sensitivity while minimizing sub-pixel bulk motion artifact [27,28]. Vascular projections over the entire imaging depth were obtained separately for each band, and on the merged extended-focus dataset. Figure 3a–c displays the individual projection images. As highlighted by the magenta arrows, the superficial vessels are most clearly resolved in the 1060 nm band projection. The cyan arrows mark the deeper vessels that are captured more uniformly by the 1550 nm imaging band. Such deeper vessels have a better contrast in the longer wavelength spectrum due to the improved signal-to-noise ratio supported by the shifted focal plane. In the 1310 nm imaging band, the vessels have a consistent but modest contrast over entire imaging depth. Figure 3d,e compare the depth-encoded colorized projection of the 1310 nm imaging subsystem to the combined projection. We note that it is difficult to appreciate the improved transverse resolution in the deeper dermis as the vessel diameter is, in general, larger in the deeper dermis compared to the papillary dermis. However, the features in the shifted focal plane imaging shown in Figure 3a,c are still manifest in the extended-focus projection.

## 5. OCT Imaging of Cleared Murine Brain

**Figure 4:**
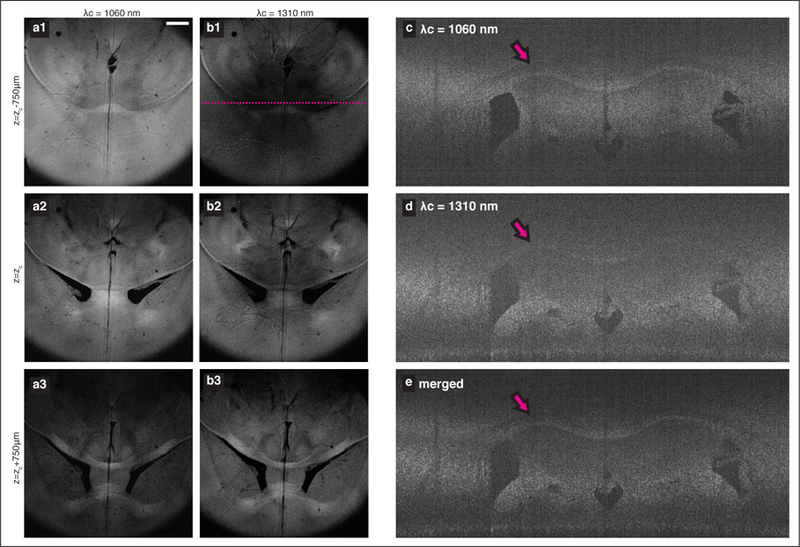
Dual-band imaging results of cleared murine brain. (**a**,**b**) Sectional MIP of two horizontal planes that are 0.75 mm apart. Comparison of a coronal section taken from (**c**) the 1060 nm band, (**d**) the 1310 nm band, and (**e**) the merged stack. Tomograms in (**c**–**e**) are taken at the line marked in (**b1**). The magenta arrows mark the corpus callosum, which is better captured by the 1060 nm system and therefore displayed with increased contrast in the merged tomogram.

The focal shift between separate imaging bands may also be useful for volumetric imaging for samples that allow larger penetration depth such as lipid-cleared tissues. It has been demonstrated that the intact brain sample can be imaged at optical resolution with clearing assisted scattering tomography (CAST) [29], a method combining OCT with a controlled clearing of lipid. While the lipid removal can be optimized to provide sufficient contrast throughout the entire depth of the sample, the shallow DoF determined by the high NA objective lens mandates step-wise adjustment of the focal plane to enable the 3D imaging for a large volume. We examined application of the multi-band source in CAST. The murine brain tissue (from a wild type C57BL/6 mouse, aged 6–8 weeks) was prepared following a customized clearing method based on SWITCH [30] protocol. Before and during imaging, the sample was submerged in a refractive-index matching solution (23.5% (*w*/*v*) *N*-methyl-D-glucamine, 29.4% (*w*/*v*) diatrizoic acid, and 32.4% (*w*/*v*) iodixanol). In general, this solution aided in suppressing scattering and achieving adequate SNR. However, the absorption loss in the 1550 nm imaging spectrum was too large to obtain a meaningful contrast, and the imaging was only performed using the two shorter wavelength subsystems. The sample was placed with superior cortex facing the objective. A set of volumetric data was acquired from individual interferometers and processed independently with depth-scale matching. A set of 29 discrete end-face sections were obtained using MIP, over a range of 105 µm for each plane. Figure 4a,b shows three selected examples of such MIP planes (horizontal cross-sections) that are 0.75 mm apart. The reduced collection efficiency due to the focal shift between imaging sources is apparent as individual sources capture different features along the depth. Figure 4c–e compares the OCT XY-tomogram (coronal section) of a single source (c) λ_*c*_ = 1060 nm and (d) λ_*c*_ = 1310 nm with the equivalent cross-section taken from the merged stack (using two bands).

## 6. Conclusions

We have demonstrated a system that leverages chromatic aberration to extend the DoF of OCT. In comparison to alternative methods, this approach does not require multiple laterally displaced beams, non-Gaussian beam optics, or complex distal microscopes. The shift in focal planes was confirmed by measurement of the transverse PSFs, and its use to extend DoF was presented in a sponge phantom, an angiographic imaging of human skin, and a cleared murine brain tissue.

The proposed method has several characteristics that should be considered when evaluating this method for a particular application. As noted previously, the sweeping bandwidth of each spectrum do not scale with a square of the center wavelength, which resulted in a mismatch in the axial resolution. We also note that this approach is suited to moderate extensions of the DoF but becomes impractical if larger extensions are required. In biological samples, including skin, the achieved transverse resolution depends both on beam focusing and on multiple-scattering, only the former of which is addressed by this technique. However, we note that this technique used the longer wavelengths, which see reduced scattering, for imaging the deeper depths. As such, it may also provide some benefit for addressing multiple-scattering degradations. Finally, it can be appreciated that the triband source constructed for this study in combination with a non-chromatically aberrating microscope can also be used to perform spectroscopic imaging.
